# Mapping the Global Landscape and Thematic Evolution of fNIRS-Gait Research: A Systematic Bibliometric and Network Visualization Analysis

**DOI:** 10.3390/bioengineering13101198

**Published:** 2026-10-15

**Authors:** Yunis Akkaş, Senem Güner, Naime Uluğ

**Affiliations:** Biomechanics and Gait Analysis Laboratory, Department of Prosthetics and Orthotics, Faculty of Health Sciences, Ankara University, Ankara 06290, Turkey; sguner@ankara.edu.tr (S.G.); naimeulug@ankara.edu.tr (N.U.)

**Keywords:** functional near-infrared spectroscopy (fNIRS), gait analysis, prefrontal cortex, dual-task walking, Mobile Brain/Body Imaging (MoBI), neurorehabilitation

## Abstract

Background: Functional near-infrared spectroscopy (fNIRS) has emerged as an essential optical neuroimaging modality in human locomotion, yet a comprehensive computational mapping of its structural and thematic evolution remains absent. This study provides a systematic, data-driven scientometric evaluation of the global fNIRS-gait research landscape across a quarter-century (2002–April 2026). Methods: A refined corpus of 348 peer-reviewed documents from the Web of Science Core Collection was computationally analyzed using VOSviewer and Bibliometrix. Results: The literature exhibits a 12.53% compound annual growth rate, shifting from stationary motor imagery toward ecologically valid overground walking. The United States leads global outputs, supported by European collaborative corridors and Chinese national infrastructures. Healthy aging (27.30%), Parkinson’s disease (10.92%), and stroke (9.20%) represent the primary clinical subdomains, centering on prefrontal cortex hyper-activation during dual-task walking (20.11%). Frontiers have migrated toward multimodal Mobile Brain/Body Imaging (MoBI) integrating virtual reality, exoskeletons, and machine learning. However, technical integration is hindered by severe signal processing heterogeneity and unstandardized short-separation channel regressions. Conclusions: Establishing standardized preprocessing pipelines and short-separation regressions may prove pivotal for transitioning mobile neuroimaging into objective, closed-loop clinical neurorehabilitation.

## 1. Introduction

Functional near-infrared spectroscopy (fNIRS) has emerged as a transformative neuroimaging modality in human movement science, offering a unique balance between portability and motion tolerance. Unlike traditional neuroimaging techniques such as functional Magnetic Resonance Imaging (fMRI) or Positron Emission Tomography (PET), which require subjects to remain stationary, fNIRS enables the monitoring of cortical activity during dynamic, overground locomotion [1]. This capability has addressed a long-standing “gap” in neuroscience, allowing researchers to explore the neural correlates of gait in ecologically valid environments—ranging from simple treadmill walking to complex real-world navigation and dual-tasking scenarios [2,3].

The significance of fNIRS in gait research is primarily rooted in its ability to capture real-time cortical engagement, particularly within the prefrontal cortex (PFC), which plays a pivotal role in the executive control of movement. Foundational studies have established that cortical activation patterns are significantly modulated by task demands, aging, and neurological conditions [1]. For instance, the growing body of “dual-task” literature underscores that walking is not merely a reflexive motor task but a cognitively demanding process that requires extensive neural resources, especially in older adults and patients with Parkinson’s disease or stroke [4,5]. Furthermore, the recent integration of fNIRS with robotic exoskeletons and Virtual Reality (VR) frameworks has opened new frontiers in neurorehabilitation, positioning fNIRS as a vital tool for developing brain-behavior biomarkers [6,7].

The specific imperative for utilizing fNIRS in human movement science is fundamentally rooted in the unique biomechanical and neurophysiological nature of upright locomotion, which renders traditional stationary neuroimaging modalities incompatible. True human gait is inherently a dynamic, multi-segmental, and gravity-defying task characterized by continuous translational acceleration of the head and body. In conventional fMRI or PET environments, such locomotor dynamics are strictly prohibited due to severe motion-related signal degradation, forcing researchers to rely on highly constrained surrogate paradigms like supine ankle dorsiflexion or motor imagery [8,9]. However, lying down or imagining a movement bypasses the complex multisensory integration—including real-time vestibular, proprioceptive, and visual flow feedback—that coordinates real overground walking [10,11]. In steady, unobstructed conditions, locomotion is largely managed by subcortical automaticity driven by spinal central pattern generators and brainstem networks [12]. Yet, when walking encounters environmental challenges (such as obstacle negotiation, sensory perturbations, or dual-task demands), the neural architecture undergoes a critical transition, shifting from subcortical automatic pathways to top-down, cognitively guided cortical control [12,13]. Because fNIRS operates via an optical method that is exceptionally tolerant to ambulatory motion thresholds, it offers the only viable neuroimaging window capable of capturing this dynamic balance between subcortical automatic pilot and cortical resource allocation during authentic, real-world human movement.

The clinical significance of fNIRS in gait research is predominantly characterized by its ability to monitor the PFC as a biomarker for compensatory cognitive control. In many gait disorders where automaticity is impaired—such as Parkinson’s disease (PD), stroke, and cerebellar ataxia—the brain recruits additional executive resources from the PFC to maintain postural stability and locomotion [14,15]. This phenomenon, often referred to as ‘cortical compensation,’ is particularly evident in patients with the Freezing of Gait (FoG) phenotype, where elevated PFC activation during both single- and dual-task walking signals a high reliance on attentional resources [13,16]. Furthermore, fNIRS studies have demonstrated that rehabilitative interventions—including treadmill training, transcutaneous vagus nerve stimulation (taVNS), and robotic assistance—can modulate these cortical patterns, leading to improved gait automaticity and reduced compensatory burden [4,17]. Understanding the temporal and task-dependent variations in PFC activity is essential for developing individualized therapeutic strategies and assessing the risk of functional decline in neurological populations [18,19]. Mechanistically, taVNS delivers low-intensity electrical stimulation to the auricular branch of the vagus nerve, which projects centrally to the nucleus tractus solitarius and, from there, to noradrenergic (locus coeruleus) and dopaminergic brainstem nuclei that modulate basal ganglia-cortical loop excitability. This autonomic-to-central pathway is the proposed physiological basis for the taVNS-related improvements in cortical activation and gait parameters reported in Parkinsonian cohorts [17].

Despite the significant technological advancements and the established clinical utility of fNIRS in gait research, the rapid expansion of the literature has introduced substantial challenges in knowledge synthesis. The current state of the field is characterized by a high degree of heterogeneity in experimental modalities, sensor configurations, and data processing pipelines, which often complicates the comparability of findings across studies [20,21]. While numerous systematic reviews have addressed specific clinical outcomes or narrow methodological aspects, there is a notable absence of a macro-scale, quantitative synthesis that maps the global intellectual landscape of the domain. This lack of standardization and the fragmented nature of cross-disciplinary collaborations—spanning engineering, rehabilitation, and clinical neurology—create a barrier to identifying emerging research hotspots and established academic hubs [22,23]. A comprehensive bibliometric analysis is required to provide a structured visualization of the field’s evolution, revealing the hidden networks of collaboration and the shifting thematic priorities over the last two decades.

To address this gap, the present study employs a rigorous bibliometric and scientometric approach to evaluate the global research output on fNIRS-based gait analysis up to 2026. By utilizing advanced science mapping tools, including VOSviewer and Bibliometrix, this research aims to (i) quantify the publication and citation trajectories to identify the most influential authors, institutions, and countries; (ii) visualize the co-authorship and co-citation networks to understand the social and intellectual structure of the field; and (iii) analyze keyword co-occurrence patterns to uncover the thematic evolution and emerging research frontiers. By providing a panoramic view of the ‘methodological landscape’ and ‘clinical focus,’ this study serves as a strategic roadmap for researchers and clinicians to navigate the rapidly growing body of evidence in mobile neuroimaging and gait rehabilitation.

## 2. Materials and Methods

Consistent with established bibliometric-mapping practice [24,25], the term “systematic” in the present study denotes a transparent, criterion-based, and reproducible literature identification and dual-reviewer screening protocol applied to a single, quality-indexed database, rather than an exhaustive multi-database systematic review conducted under a PRISMA-type framework. The scope and rationale of this design choice are detailed in Section 2.1 and revisited as a study limitation in Section 4.4.

### 2.1. Data Source and Search Strategy

**Figure 1:**
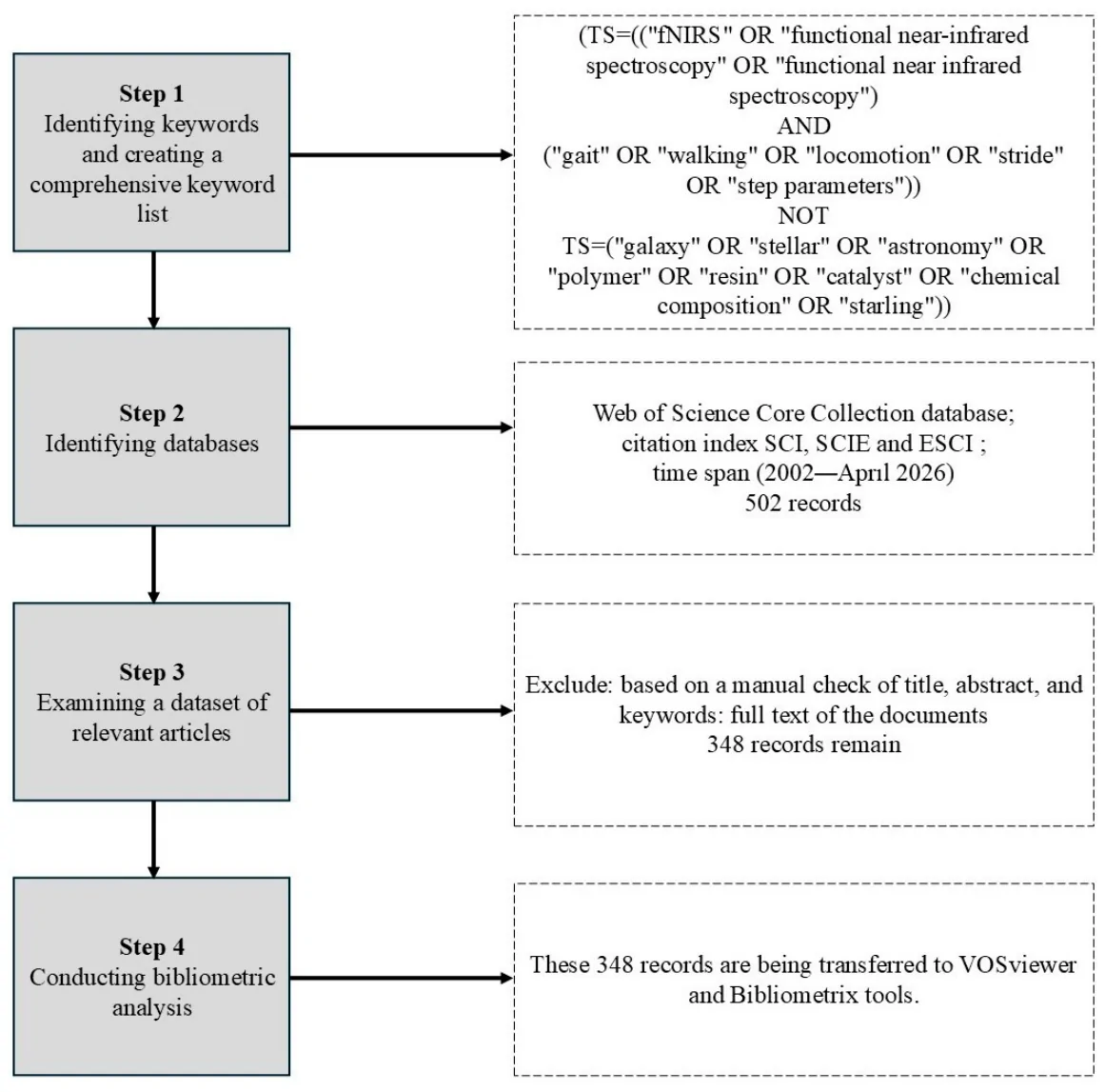
The process of this research.

The literature retrieval and filtration workflow for this study was systematically executed to ensure transparency, reproducibility, and rigorous data quality, as illustrated in the flow diagram (Figure 1). The Web of Science Core Collection (WoSCC) was utilized as the primary database source, with the search restricted to high-impact citation indexes including the Science Citation Index (SCI), Science Citation Index Expanded (SCI-EXPANDED), and the Emerging Sources Citation Index (ESCI). The temporal boundary for the literature mapping spanned from 2002 to April 2026. To capture the exact intersection of neuroimaging and locomotion sciences, a comprehensive search string was constructed using Boolean operators to bridge fNIRS-related concepts with specific gait parameters, while explicitly implementing negative filters (NOT) to eliminate homonymous or unrelated terms from fields such as astronomy and polymer chemistry (Figure 1). For full transparency and reproducibility, the exact Boolean query executed in the WoSCC “Topic” field in April 2026 was: (TS = ((“fNIRS” OR “functional near-infrared spectroscopy” OR “functional near infrared spectroscopy”) AND (“gait” OR “walking” OR “locomotion” OR “stride” OR “step parameters”)) NOT TS = (“galaxy” OR “stellar” OR “astronomy” OR “polymer” OR “resin” OR “catalyst” OR “chemical composition” OR “starling”)), restricted to the document types “Article” and “Review” published in English. The WoSCC was deliberately selected as the sole data source because its uniformly curated indexing and citation-linkage structure is a methodological prerequisite for the citation-based performance metrics and co-citation network reported in Section 3.5 and Section 3.6; merging records from heterogeneously structured databases (e.g., Scopus, PubMed, IEEE Xplore) without a validated cross-database deduplication and reference-matching protocol would risk introducing citation-count artifacts into these analyses. We fully acknowledge in Section 4.4 that this single-database strategy may under-represent the engineering-oriented technical literature indexed primarily outside WoSCC.

The automated database execution using this finalized query yielded an initial pool of 502 records. A two-stage manual screening protocol was subsequently performed independently by two researchers to purify the dataset and eliminate peripheral literature. In the first stage, the titles, abstracts, and author keywords of all 502 records were reviewed against the inclusion and exclusion criteria defined below to exclude irrelevant publications; in the second stage, full texts were examined whenever eligibility remained uncertain. Studies that did not directly measure, evaluate, or model cortical activity in relation to human gait metrics or clinical rehabilitation frameworks were excluded, and disagreements between the two reviewers were resolved through discussion and consensus. Following this manual curation phase, a refined corpus of 348 core records remained (Figure 1). These 348 records were exported from WoSCC in full-record and cited-reference format (.txt and .bib) and subsequently imported into VOSviewer (v1.6.20) and Bibliometrix (v5.4.0, via the R-based(2026.05.1) Biblioshiny interface) to perform, respectively, network science mapping and macro-level performance-metric evaluation [24,25].

The inclusion criteria were:

- Studies involving fNIRS;
- Studies evaluating human gait, walking, locomotion, or mobility-related outcomes;
- Original articles and review papers published in peer-reviewed journals;
- Publications written in English.

The exclusion criteria were:

- Conference abstracts, editorials, letters, notes, and book chapters;
- Animal studies;
- Studies unrelated to gait or locomotion;
- Publications focusing on non-biological applications of near-infrared spectroscopy.

Following the screening procedure, 348 records met the eligibility criteria and were included in the final bibliometric analysis.

### 2.2. Bibliometric Tools and Data Analysis

To comprehensively analyze and visualize the structural, social, and conceptual frameworks of the retrieved fNIRS-gait literature, two specialized scientometric software suites were utilized. The raw dataset containing 348 core records was exported from the Web of Science database and processed chronologically through the following analytical pipelines:

- Performance and Strategic Mapping (Bibliometrix v5.4.0): Quantitative productivity metrics were compiled using the open-source, R-based (v5.4.0, via the R-based(2026.05.1) Biblioshiny interface) Bibliometrix software via the Biblioshiny web interface. This application was utilized to aggregate descriptive statistics, including annual scientific production trajectories, top-performing countries, institutional affiliations, and source journal distributions. Furthermore, Callon’s centrality and density metrics were calculated based on keyword co-occurrences to establish the quadrants of the strategic Thematic Map. Association-strength normalization was selected as the network weighting method because it is widely recommended for bibliometric mapping and facilitates the identification of meaningful relationships among authors, institutions, references, and keywords.
- Network Visualization and Clustering (VOSviewer v1.6.20): Science mapping and distance-based network visualizations were executed using VOSviewer. Fractional counting was systematically applied to eliminate size-bias artifacts among multi-authored and multi-institutional papers. To uncover collaborative patterns and thematic hotspots, co-authorship networks (for countries and institutions) and keyword co-occurrence maps were constructed. For the keyword co-occurrence network, a minimum occurrence threshold of 6 per author keyword and a minimum cluster size of 1 were implemented (association-strength normalization, VOSviewer resolution parameter = 1.00) to optimize readability and filter out peripheral, low-frequency terminologies, retaining only consolidated research concepts. Finally, these keyword structures were synthesized alongside the thematic map to trace how the field has shifted from basic motor imagery protocols toward complex, real-world dual-task and neurorehabilitation setups.
- To improve data consistency and network interpretability, a data harmonization procedure was performed prior to visualization. Synonymous keywords, spelling variations, and abbreviation-based duplicates (e.g., “fNIRS” and “functional near-infrared spectroscopy”) were manually reviewed and unified when necessary (e.g., plural/singular variants such as “exoskeleton”/“exoskeletons”, British/American spelling variants, and acronym-expansion pairs such as “VR”/“virtual reality” and “BCI”/“brain–computer interface” were merged into a single controlled term prior to network construction; the complete harmonization thesaurus is available from the corresponding author upon request). Network maps were generated using association-strength normalization (VOSviewer resolution parameter = 1.00); for the keyword co-occurrence network a minimum threshold of 6 occurrences per author keyword and a minimum cluster size of 1 were applied, and for the country- and institution-level co-authorship networks a minimum threshold of 5 documents per unit was applied; nodes and links falling below these thresholds were excluded to reduce fragmentation and improve cluster interpretability. These procedures were implemented to reduce fragmentation, improve cluster stability, and enhance the interpretability of collaboration and thematic networks.

## 3. Results

To maintain high thematic precision, the initial 502 search results were subjected to a dual-expert review, resulting in a final corpus of 348 high-quality articles. The bibliometric outcomes provided below offer a multi-dimensional perspective on the global publication trends, collaborative networks, and the shifting research focus within the fNIRS-based gait research community.

The bibliometric landscape of fNIRS applications in gait analysis was evaluated based on a refined dataset of 348 documents spanning from 2002 to 2026. These documents were published across 141 different sources, indicating a diverse academic interest in the field. The dataset shows a robust Annual Growth Rate of 12.53%, reflecting the increasing adoption of portable neuroimaging technologies in locomotion research.

On average, the analyzed documents have a mean age of 5.17 years, suggesting that the majority of the influential work has been produced recently. The scientific impact of the collection is significant, with an average of 38.66 citations per document. A total of 1346 authors contributed to this body of knowledge, with a high degree of collaboration, as evidenced by an average of 6.11 co-authors per document and an international co-authorship rate of 29.02%. Regarding document types, the vast majority of the records are original research articles (*n* =324), followed by reviews (*n* = 10), ensuring that the analysis is primarily based on primary empirical data.

### 3.1. Annual Publication Trends

**Figure 2:**
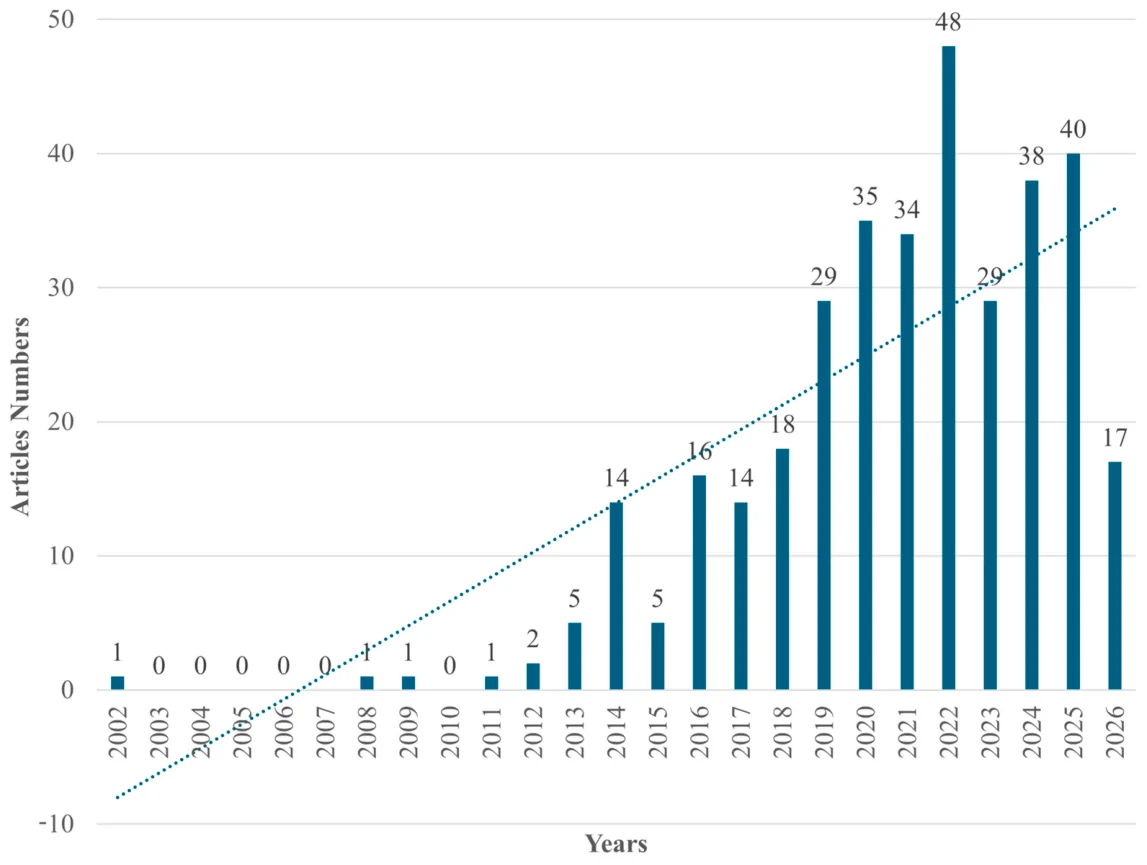
Annual scientific production of fNIRS-based gait analysis research (2002–2026).

The chronological distribution of the 348 selected documents, spanning from 2002 to early 2026, reflects a dynamic and rapidly evolving research field (Figure 2). During the initial decade (2002–2012), the scientific output remained limited and sporadic, representing the pioneering stage of fNIRS applications in gait analysis. However, a significant upward shift in publication volume is observed following 2014.

From 2015 onwards, the field entered a phase of sustained growth, which became particularly pronounced between 2020 and 2024. This period accounts for the highest density of annual scientific production in the dataset. The robust Annual Growth Rate of 12.53% substantiates the intensifying academic interest in monitoring cortical activity during human locomotion. While the data for 2025 and 2026 are still accumulating, the current trajectory indicates that the integration of fNIRS into gait research has transitioned from an experimental niche to a mainstream methodological approach.

### 3.2. Analysis by Country/Region & Collaboration World Map

**Figure 3:**
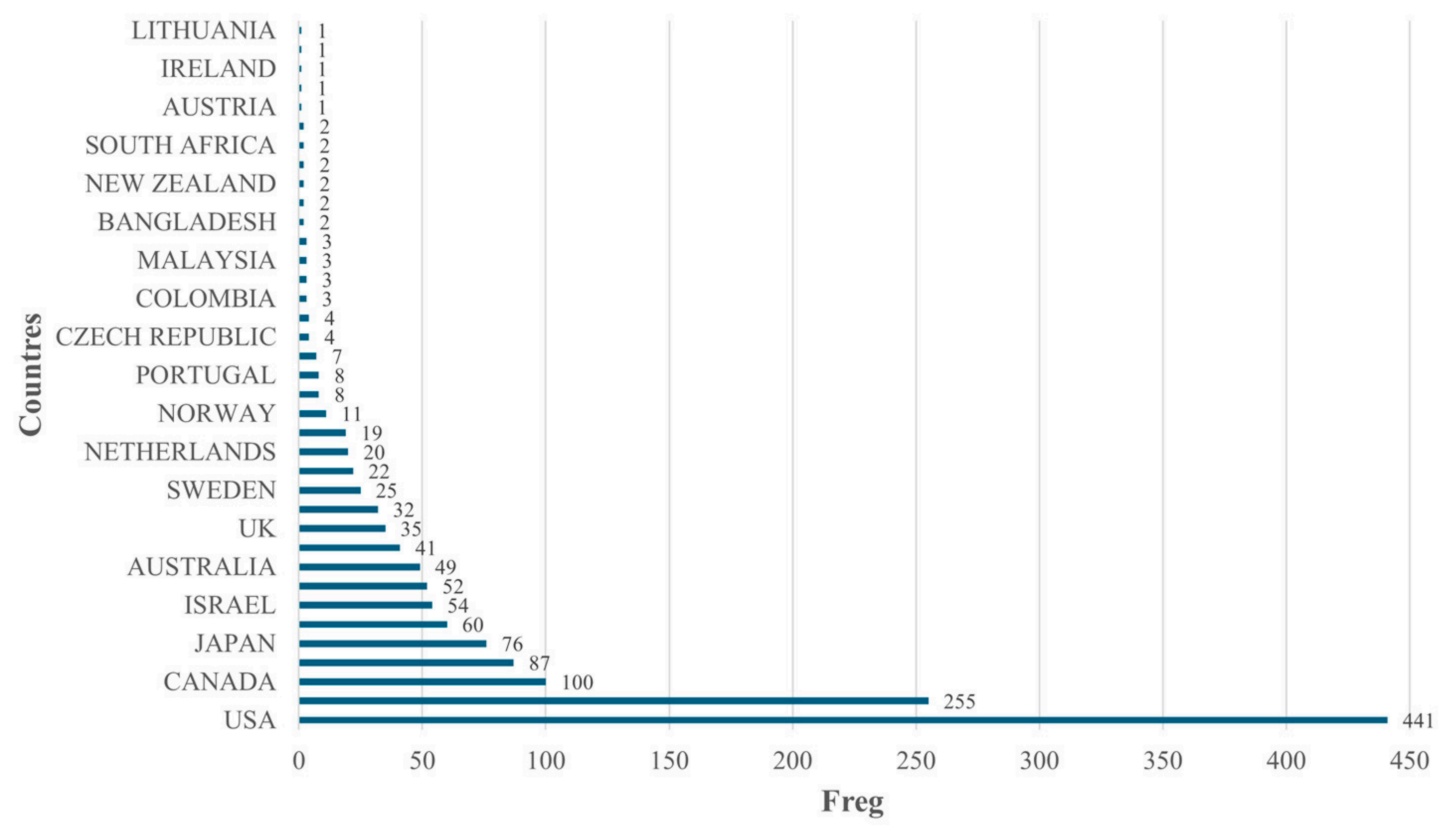
Most productive countries in fNIRS-based gait research. SCP: Single Country Publications; MCP: Multiple Country Publications (Frequency denotes the total number of articles based on corresponding author affiliation).

The geographical distribution of 348 documents highlights a global interest in fNIRS-based gait analysis, led by a few highly productive nations. Figure 3 presents the top 10 most productive countries, categorized by Single Country Publications (SCP) and Multiple Country Publications (MCP). In this analysis, ‘Frequency’ (Freq) represents the total number of articles attributed to a specific country based on the affiliation of the corresponding author. This metric provides a standardized measure of a nation’s primary research output and leadership in the field.

According to the results, the USA leads the field with the highest total frequency, followed by China and Germany. A notable finding is the high ratio of MCP in countries such as Germany or UK, indicating a strong emphasis on international collaboration. Conversely, China shows a substantial volume of SCP, reflecting a robust intra-national research infrastructure.

**Figure 4:**
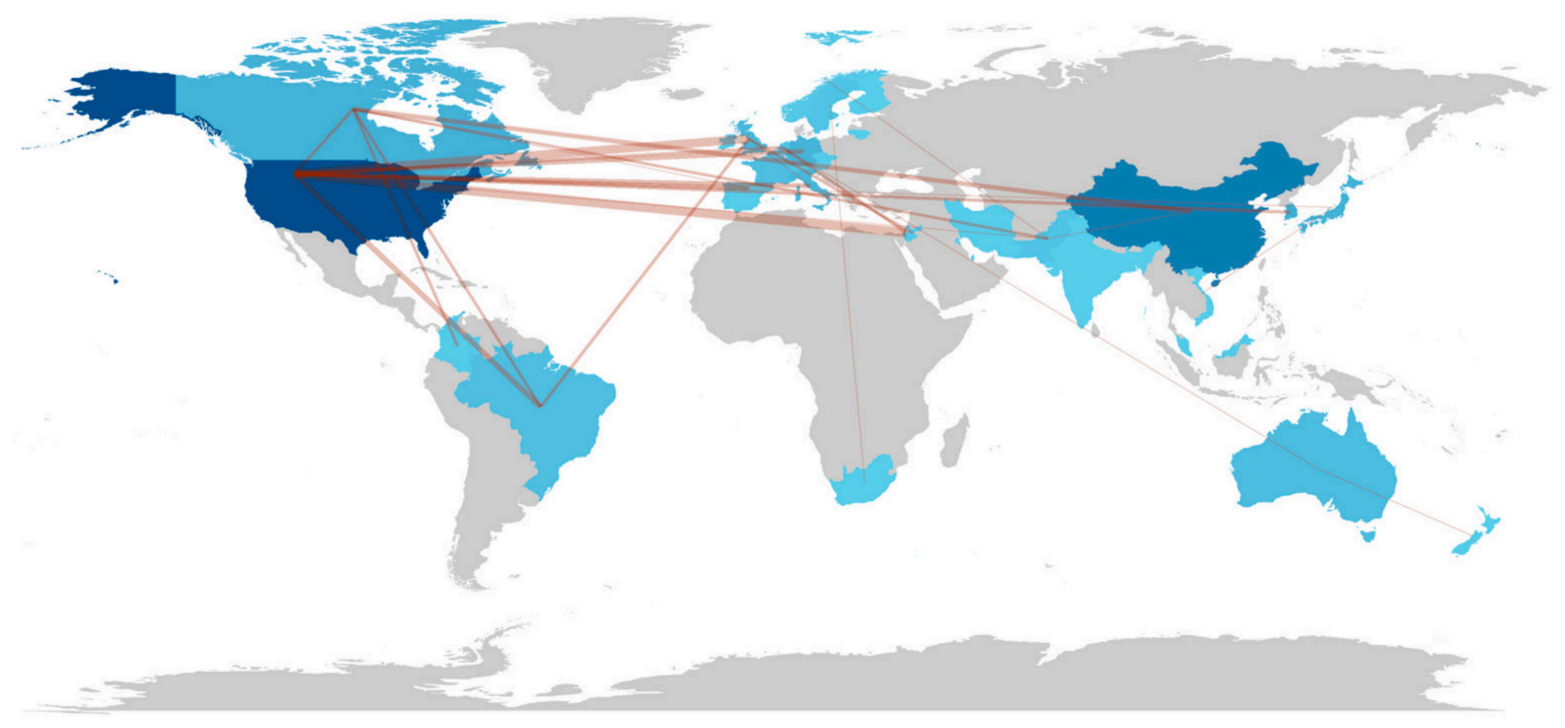
Country collaboration world map. The intensity of blue represents publication volume, with darker blue indicating a higher number of publications and lighter blue indicating a lower number of publications. Lines indicate international research collaborations between countries.

To further visualize these scientific networks, a Collaboration World Map was generated (Figure 4). The map illustrates the intensity of research activities across different continents, with the darkest blue regions representing the highest publication counts. The connecting lines represent active research corridors, revealing significant international partnerships between the USA, Europe, and East Asia. This global network underlines that fNIRS-based gait research is not localized but rather a collaborative transdisciplinary effort aimed at solving complex problems in human movement science.

### 3.3. Analysis by Institution

**Figure 5:**
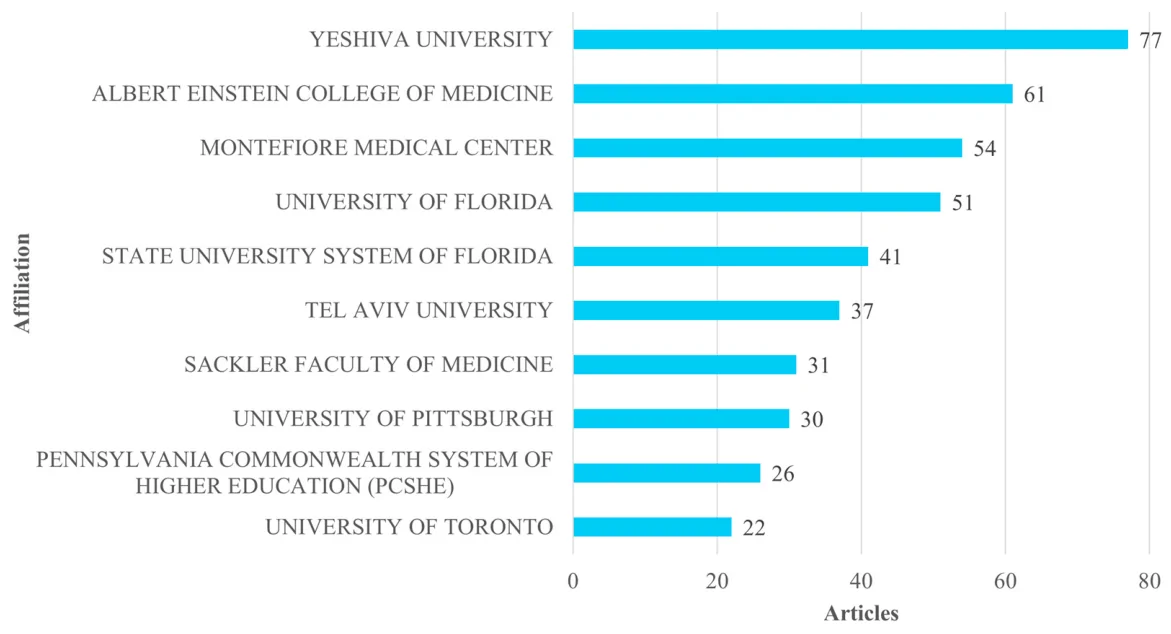
Top 10 most productive institutions in fNIRS-based gait analysis research (2002–2026). The ranking is based on the total number of articles attributed to each affiliation.

The institutional analysis provides insights into the major academic centers contributing to the development of fNIRS-based gait research. A total of 582 institutions were identified across the 348 analyzed publications. Figure 5 presents the most productive affiliations based on the total number of publications.

The results indicate that Yeshiva University emerged as the most productive institution in the field, followed by Albert Einstein College of Medicine and Montefiore Medical Center. These institutions form a highly interconnected research ecosystem that has substantially contributed to the understanding of cognitive-motor interactions, aging-related mobility decline, and cortical control of gait. Other highly productive institutions included the University of Florida, the University of Michigan, Drexel University, Humboldt University, and the University of Genoa, reflecting the broad international distribution of expertise in mobile neuroimaging and locomotion research.

The dominance of several institutions from the United States highlights the country’s leading role in the field and mirrors the findings of the country-level analysis. At the same time, the presence of European institutions among the most productive affiliations demonstrates the increasing contribution of international research networks. Collectively, these institutions appear to function as major scientific hubs that drive methodological innovation, interdisciplinary collaboration, and clinical translation in fNIRS-based gait analysis research.

### 3.4. Analysis by Authors

**Figure 6:**
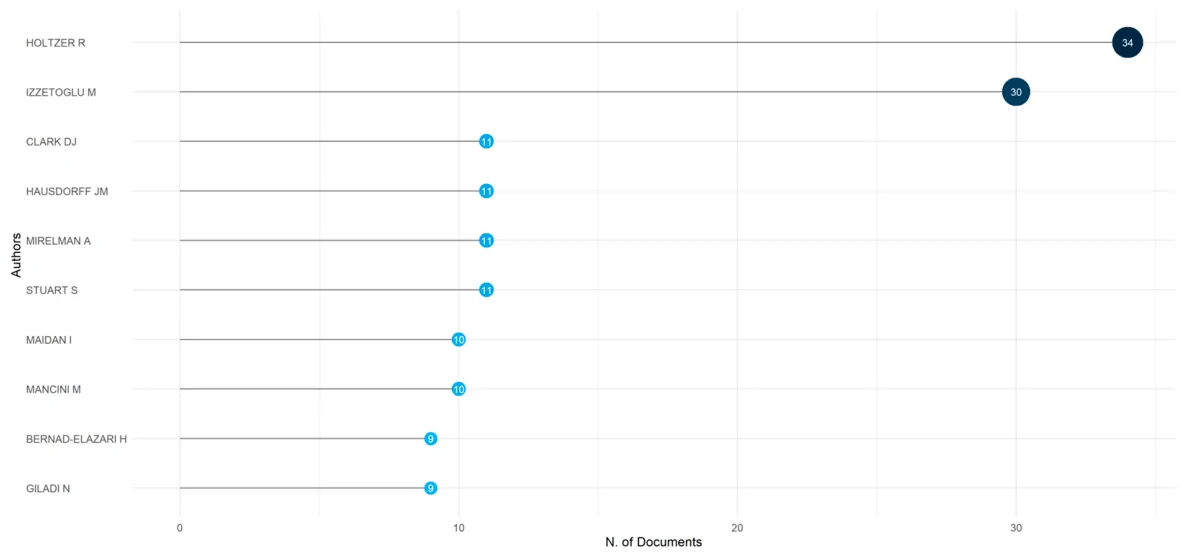
Top 10 most relevant authors based on the number of publications.

The analysis of authors provides a comprehensive look at the leading researchers who have shaped the trajectory of fNIRS applications in gait analysis. A total of 1346 authors contributed to the analyzed documents. Figure 6 illustrates the top 10 most relevant authors based on their total publication volume. Holtzer R., Izzetoglu M., and Perrey S. emerge as the most prolific contributors, demonstrating a sustained commitment to the field.

**Figure 7:**
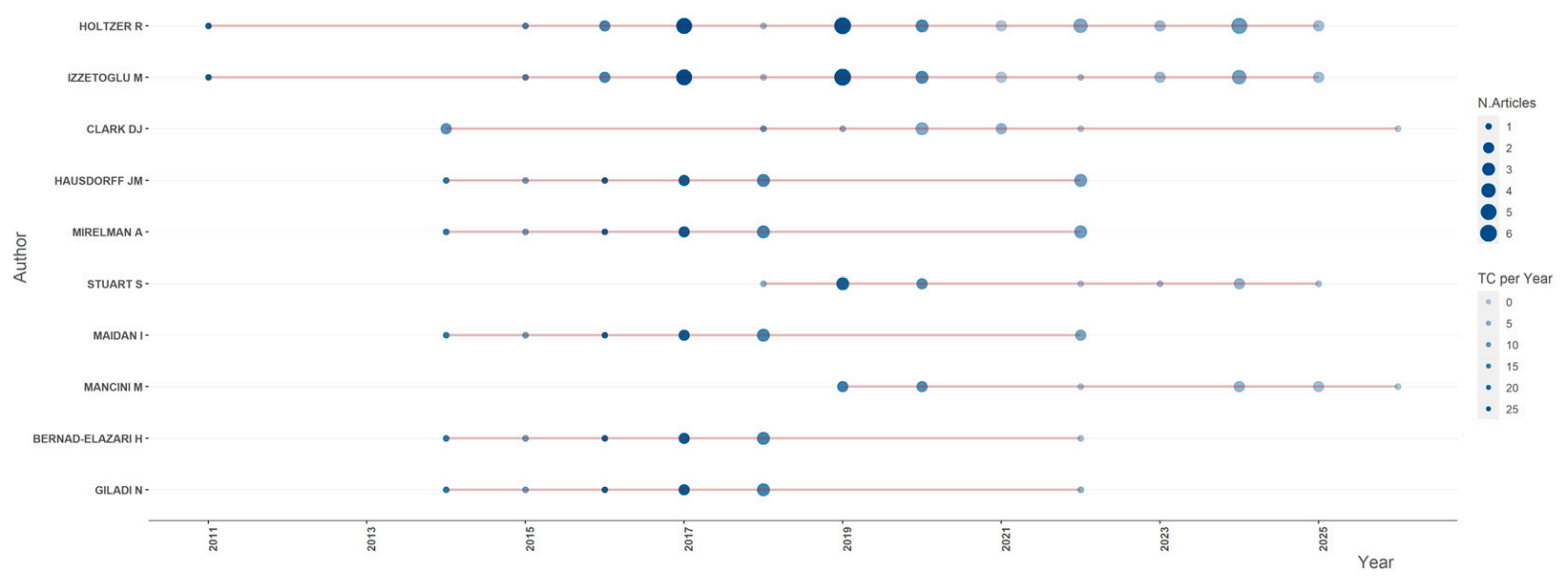
Authors’ production over time (Circle size indicates the number of articles; color intensity indicates total citations per year).

To further evaluate the consistency and impact of these researchers, Figure 7 displays the authors’ production over time. The size of the circles represents the number of papers per year, while the color intensity reflects the citation impact. This visualization reveals that authors like Holtzer R. and Izzetoglu M. have maintained a steady research output for over a decade, whereas some authors have shown a significant increase in research activity in recent years, aligning with the overall growth of the field.

**Figure 8:**
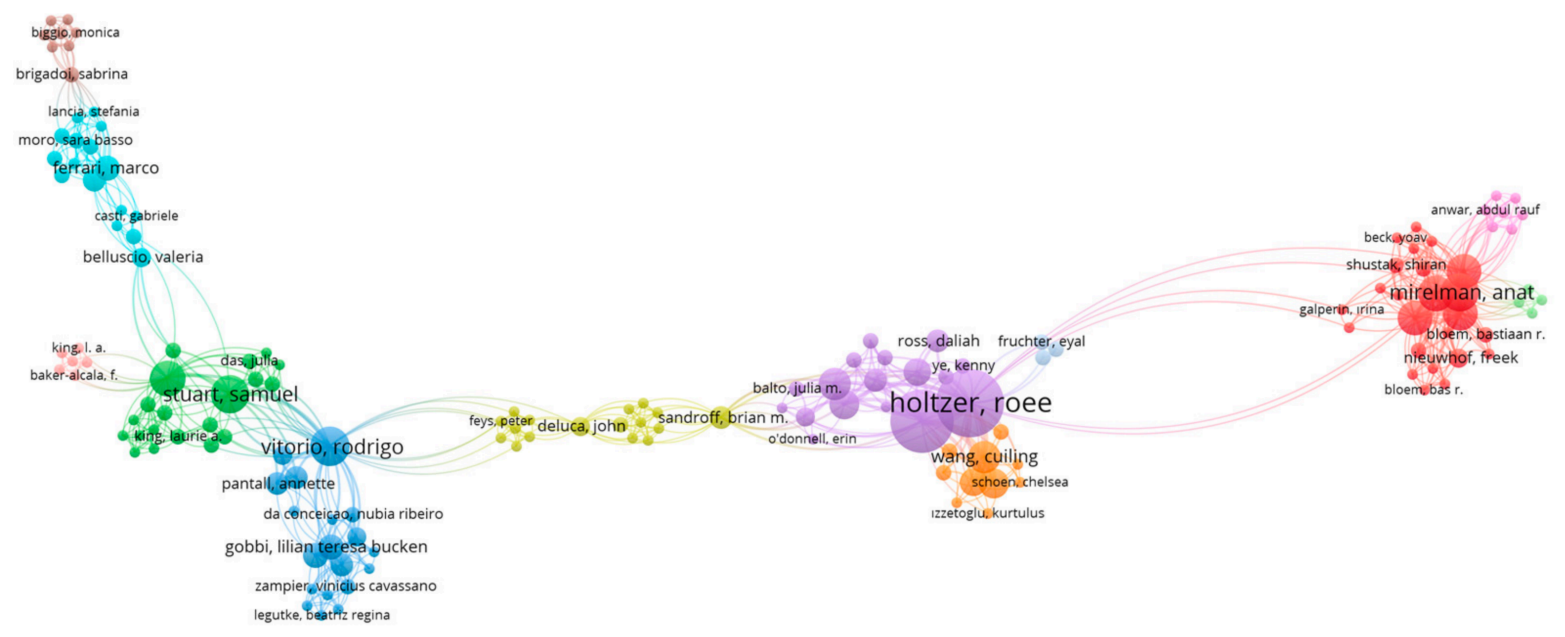
Co-authorship network visualization of researchers in fNIRS-based gait analysis. Nodes represent authors, and links represent collaborative publications.

Beyond individual productivity, the social structure of the research community was analyzed using VOSviewer (Figure 8). The co-authorship network reveals several distinct research clusters (represented by different colors). The largest clusters are centered around influential figures such as Holtzer R. and Verghese J., indicating strong collaborative ties in neurorehabilitation and cognitive-motor interference studies. The density of connections within these clusters suggests a high level of knowledge exchange and interdisciplinary cooperation, which is essential for managing the technical and clinical complexities of fNIRS-based gait research.

### 3.5. Analysis of the Journals and Cited Journals

The 348 documents included in this bibliometric analysis were published across 141 different journals, reflecting the multidisciplinary nature of fNIRS-based gait research and its intersection with neuroscience, rehabilitation, geriatrics, biomedical engineering, and movement science. Table 1 presents the most productive journals in the field based on publication output, citation performance, and bibliometric impact indicators.

Among the identified sources, *Frontiers in Aging Neuroscience* emerged as the most productive journal, publishing 18 articles related to fNIRS-based gait analysis. It was followed by *Frontiers in Human Neuroscience* and *Neurorehabilitation and Neural Repair*, each contributing 15 publications. Other major publication venues included the *Journal of NeuroEngineering and Rehabilitation*, *NeuroImage*, *Frontiers in Neurology*, *IEEE Transactions on Neural Systems and Rehabilitation Engineering*, *Sensors*, and *Gait & Posture*. The distribution of journals highlights the interdisciplinary character of the field, which integrates neuroimaging methodologies with clinical rehabilitation and human movement analysis.

Citation-based indicators revealed a slightly different pattern. Although *Frontiers in Aging Neuroscience* published the largest number of articles, journals such as *NeuroImage*, *Gait & Posture*, and *Frontiers in Human Neuroscience* demonstrated particularly strong citation influence within the research network. Notably, *NeuroImage* exhibited the highest local citation count, indicating its central role in establishing methodological and theoretical foundations for fNIRS-related neuroimaging research.

**Figure 9:**
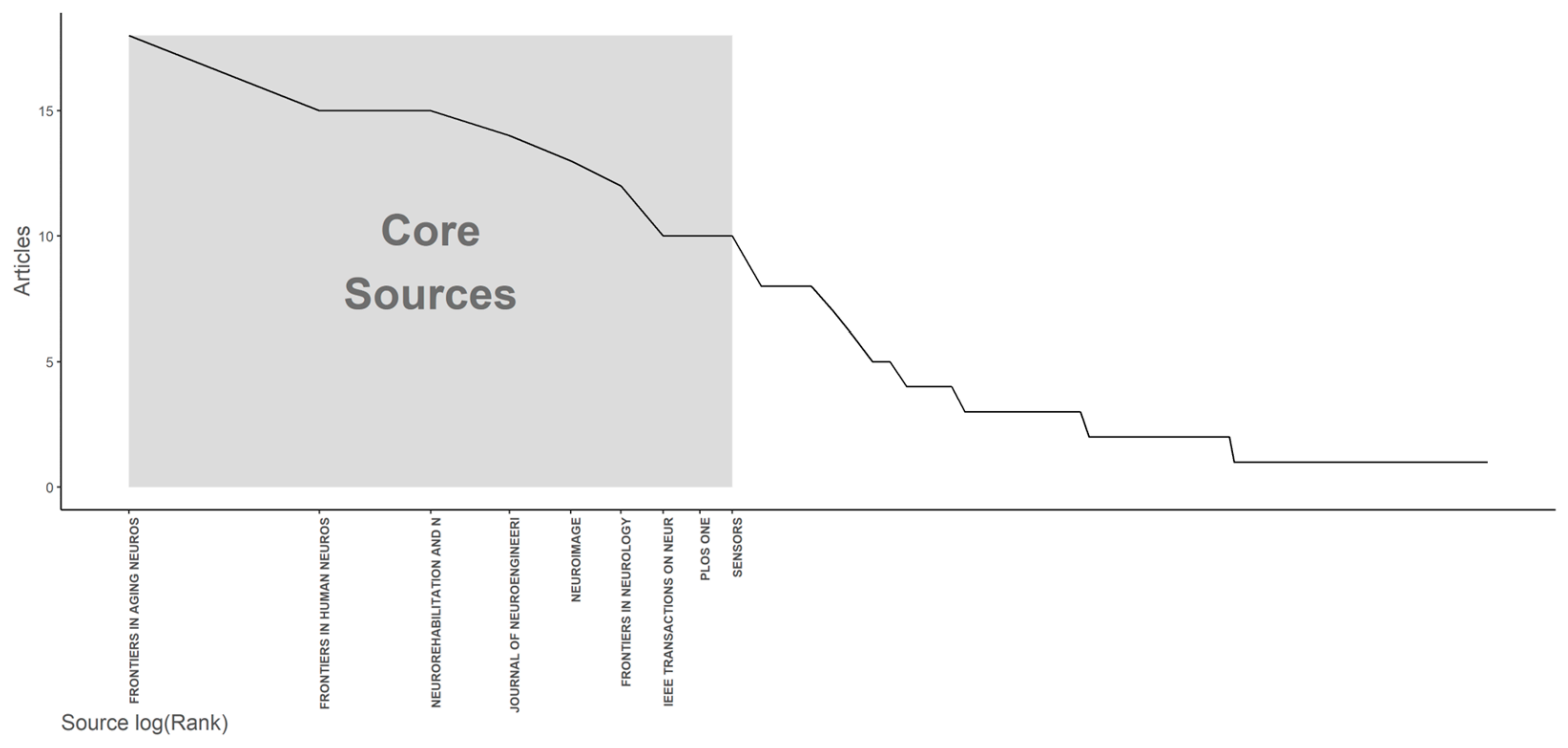
Bradford’s Law visualization showing the core journals (Zone 1) in the research field.

To further identify the core publication sources within the field, Bradford’s Law of Scattering was applied (Figure 9). The analysis demonstrated that a relatively small group of journals accounted for a disproportionately large share of the published literature. These core journals collectively produced approximately one-third of all publications included in the dataset, confirming a concentration of scientific output within a limited number of highly influential outlets. This pattern is characteristic of a maturing research domain in which specialized journals become the primary channels for disseminating methodological innovations and clinically relevant findings.

According to Bradford’s Law, journals are divided into three zones (Zone 1, 2, and 3) based on their publication frequency. Zone 1 represents the ‘core’ journals of the field. In this study, the analysis identified a small group of journals as the nucleus of the research area. These core journals account for one-third of the total publications, suggesting that despite the wide variety of sources, the most influential research is highly concentrated in a few specialized outlets. This concentration is a typical characteristic of a rapidly maturing scientific field where specific high-impact journals become the primary platforms for disseminating new findings.

### 3.6. Intellectual Structure of the Field: Reference Co-Citation Analysis

**Figure 10:**
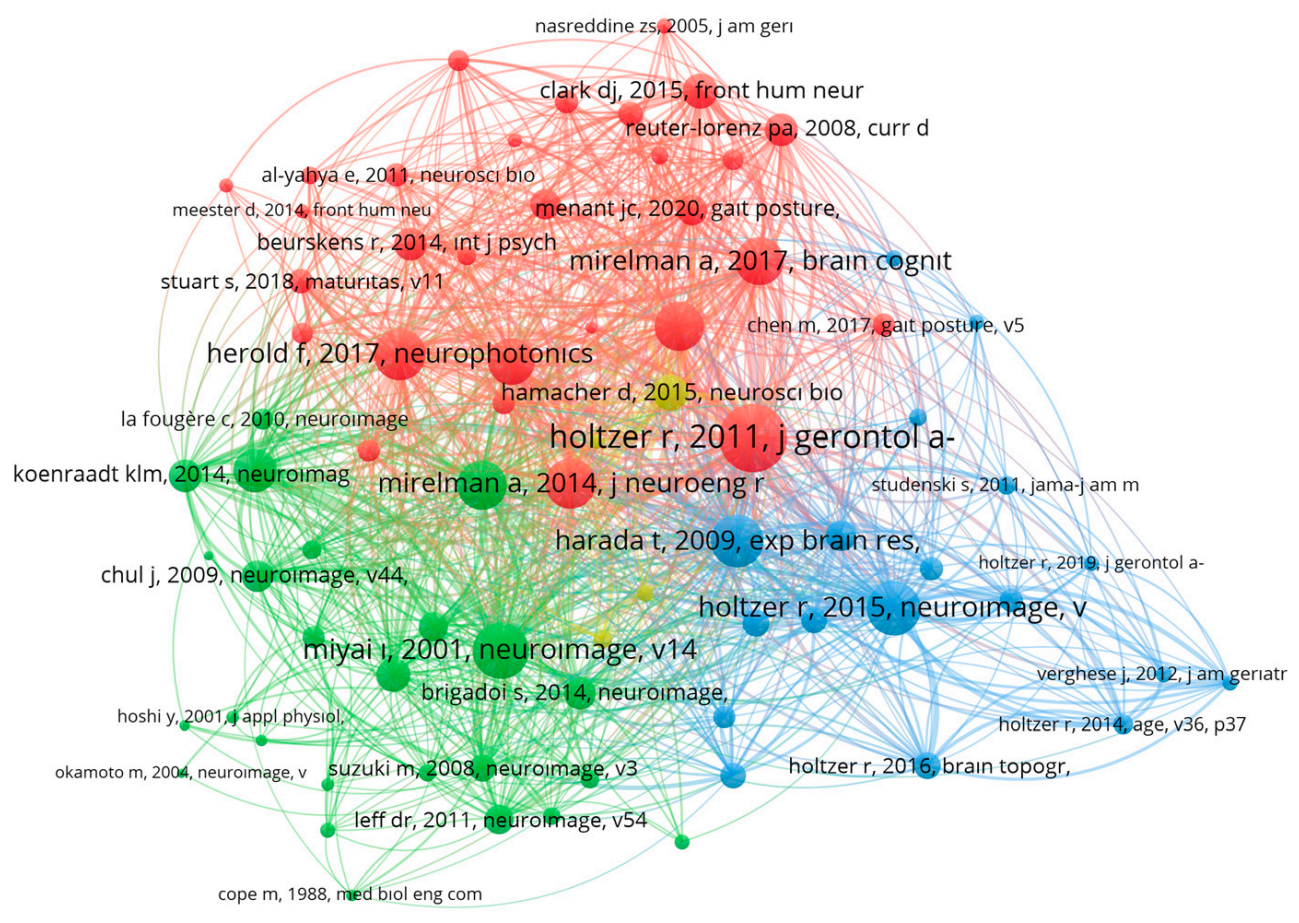
Co-citation network of cited references. The size of the nodes represents the citation frequency. Different colors indicate distinct thematic clusters of the references. The connecting lines represent co-citation relationships between them.

The analysis of cited references identifies the foundational knowledge and landmark studies upon which current fNIRS-based gait research is built. Out of the 10,948 references extracted from the dataset, a co-citation network was generated using VOSviewer to visualize the intellectual structure of the field (Figure 10).

The network reveals several distinct clusters of research, with highly influential publications acting as central nodes. Landmark studies such as Holtzer et al. (2011), which focused on cognitive-motor interference, and Suzuki (2008), regarding neurovascular coupling, emerge as the primary pillars of the literature [1,26]. Additionally, the foundational work of Miyai et al. (2002) on cortical activation during gait serves as a common reference across different clusters [27]. 

Instead of a fragmented literature, the co-citation map demonstrates a high degree of integration between clinical neurology and biomechanical engineering. The proximity of these key references in the network suggests that the field has evolved from early feasibility studies toward complex investigations of brain-behavior relationships during ecological walking. These ‘foundational’ documents continue to receive steady citations, acting as the theoretical framework for modern innovations in wireless fNIRS and real-world gait monitoring.

### 3.7. Thematic Evolution and Keyword Co-Occurrence Analysis

**Figure 11:**
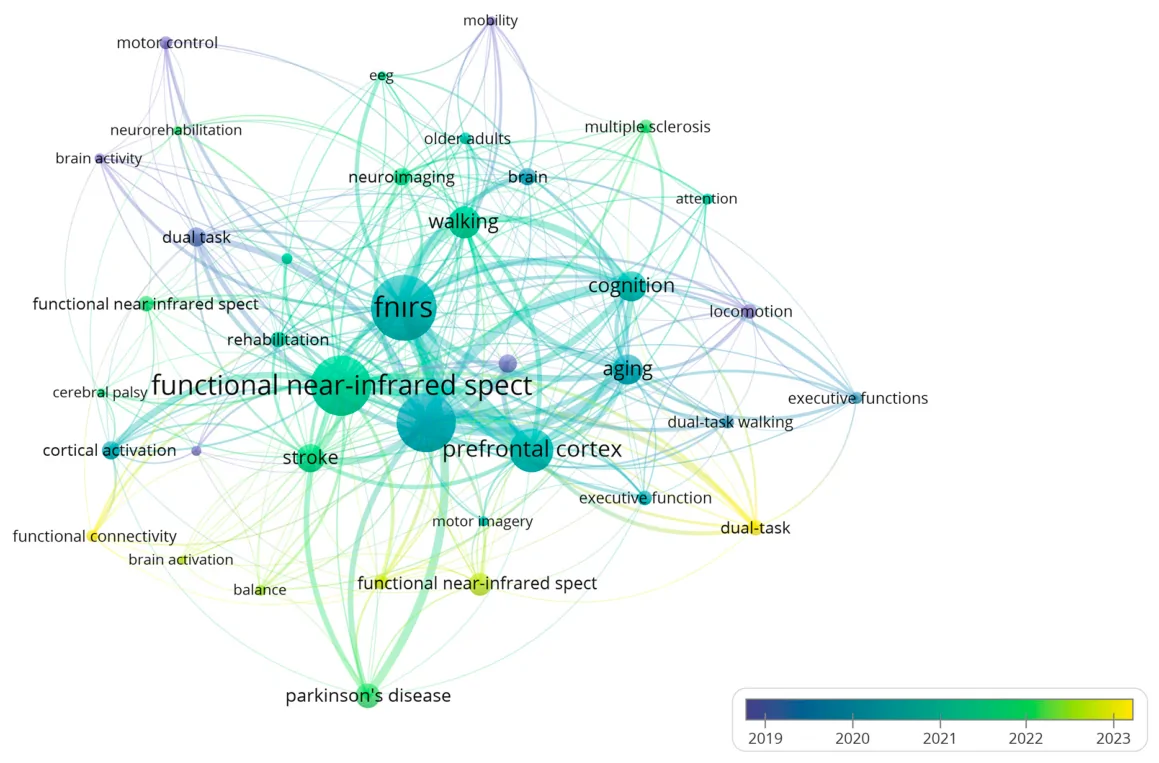
Network visualization of author keyword co-occurrence. The size of the nodes represents the frequency of the keywords, the thickness of the lines indicates the strength of the co-occurrence, and the colors indicate the average publication year associated with each keyword.

The conceptual evolution of the field was analyzed using an overlay visualization to identify the temporal distribution of research themes (Figure 11). In this map, the color of the nodes represents the average publication year of the documents in which each keyword appears. The temporal scale (2019 to 2023) represents the weighted average period when the majority of the analyzed keywords gained significant academic traction, rather than the total timespan of the dataset (2002–2026). This specific interval is highlighted because it encompasses the most intensive phase of research activity and the emergence of modern methodological standards.

- Long-Standing Core Themes (Purple/Dark Blue): Early core keywords such as ‘fNIRS’, ‘walking’, ‘motor cortex’, and ‘prefrontal cortex’ appear in darker tones. These nodes represent the foundational neuroimaging studies that established the feasibility of monitoring cortical activity during locomotion.
- Established Research Paradigms (Green/Teal): Terms like ‘older adults’, ‘dual-task’, ‘executive function’, and ‘Parkinson’s disease’ are positioned in the middle of the temporal scale (approx. 2021). This indicates that cognitive-motor interference and clinical gait studies became the central and established pillars of the field during this period.
- Emerging Fronts (Yellow): The most recent research trends (2022–2023+) are represented by yellow-coded nodes, including ‘virtual reality’, ‘exoskeletons’, ‘machine learning’, and ‘brain–computer interface’. The shift toward these keywords suggests a recent focus on integrating fNIRS with immersive technologies and advanced computational models.

Quantitatively, the transition of colors from the center towards the periphery indicates that the field has evolved from basic neural mapping toward complex, technology-driven applications in ecologically valid environments.

### 3.8. Thematic Map

**Figure 12:**
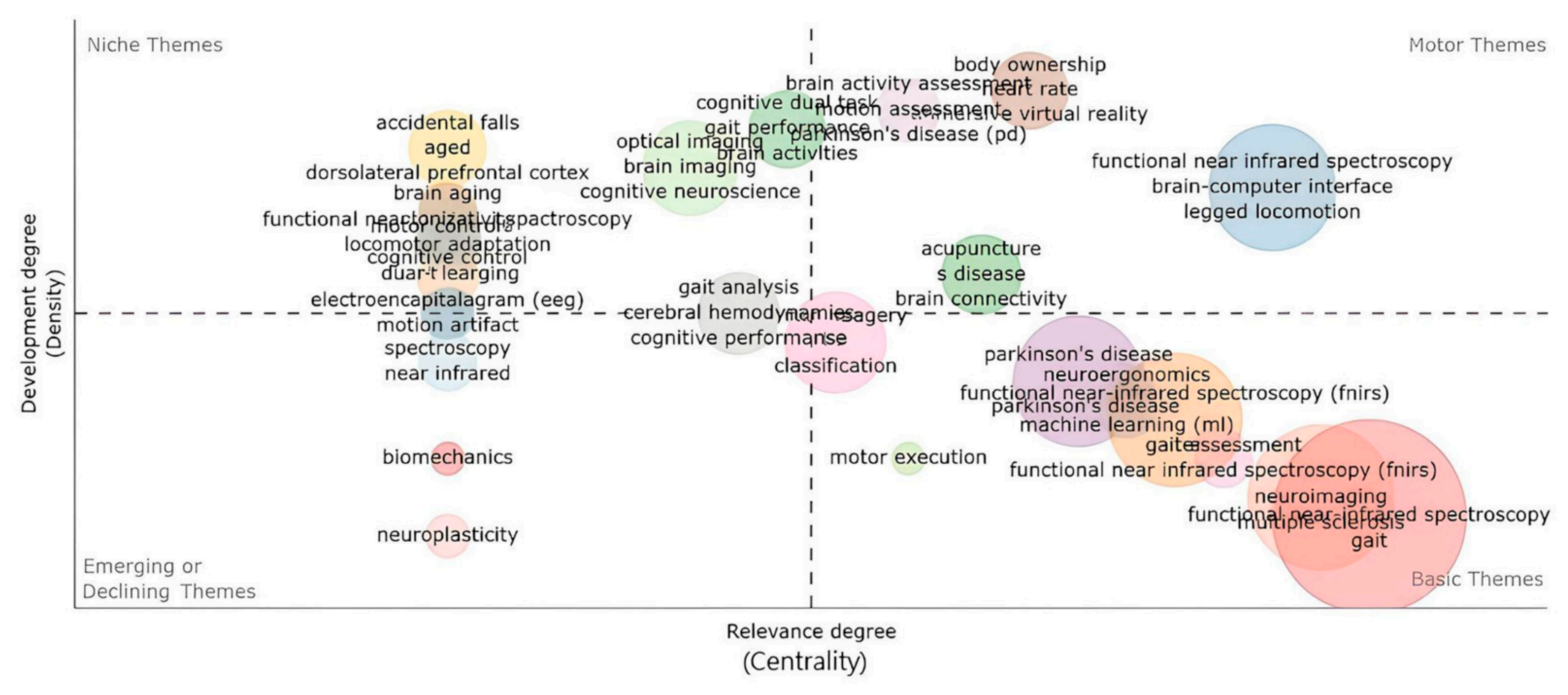
Thematic map of fNIRS-based gait analysis research. The quadrants classify keywords as Motor (developed/central), Basic (foundational/central), Niche (developed/isolated), and Emerging/Declining (peripheral).

To evaluate the strategic position and development level of the research themes, a Thematic Map was generated based on Callon’s density (*y*-axis) and centrality (*x*-axis) measures (Figure 12). The map categorizes keywords into four quadrants, providing a visualization of the conceptual maturity of the field:

- Motor Themes (Upper-Right Quadrant): These are well-developed and essential themes that drive the research field. This quadrant features prominent topics such as ‘functional near-infrared spectroscopy’, ‘brain–computer interface’, ‘legged locomotion’, and ‘immersive virtual reality’. The presence of these terms indicates that the integration of fNIRS with BCIs and VR is currently a highly developed and central research pillar.
- Basic Themes (Lower-Right Quadrant): These represent fundamental topics that are highly relevant to the field but require further specialized development. The largest cluster in this area is centered on ‘gait’, ‘neuroimaging’, ‘multiple sclerosis’, and ‘fnirs’. Other clusters include ‘parkinson’s disease’, ‘machine learning’, and ‘neuroergonomics’. These themes serve as the foundational building blocks of the current literature.
- Niche Themes (Upper-Left Quadrant): These are highly specialized and developed topics with limited centrality to the overall field. This quadrant includes terms like ‘accidental falls’, ‘dorsolateral prefrontal cortex’, ‘brain aging’, and ‘locomotor adaptation’. While technologically or clinically advanced, these topics represent specific sub-specializations within the broader gait research community.
- Emerging or Declining Themes (Lower-Left Quadrant): This quadrant identifies themes that are either emerging or losing momentum. Topics such as ‘biomechanics’ and ‘neuroplasticity’ are located here, suggesting that while they contribute to the field, they are currently on the periphery compared to the dominant neuroimaging-centered themes.

The distribution of the clusters indicates a field that is strongly rooted in clinical gait analysis and neuroimaging (Basic Themes) but is rapidly evolving toward sophisticated technological interfaces like VR and machine learning (Motor Themes).

### 3.9. Dual-Task and Clinical Population Analysis

A targeted analysis of the dataset was conducted to determine the prevalence of specific experimental paradigms and clinical focuses within the 348 analyzed documents. The results indicate that Dual-Task (DT) walking is the primary experimental protocol, appearing in 20.11% (*n* = 70) of the studies. This confirms fNIRS as a leading tool for assessing cognitive-motor interference during complex walking conditions. Frequencies were calculated based on keyword occurrence and thematic categorization of the retrieved records. Because individual publications could contribute to more than one thematic category, percentages should be interpreted as thematic prevalence rather than mutually exclusive study classifications.

The analysis of clinical and demographic populations reveals a clear hierarchy:

- Aging and Geriatrics: Research involving older adults and aging represents the largest segment of the literature, identified in 27.30% (*n* = 95) of the documents. These studies primarily investigate age-related neural changes, frailty, and fall risk.
- Neurological Disorders: Among clinical populations, Parkinson’s Disease is the most frequently studied condition (10.92%, *n* = 38), followed by Stroke (9.20%, *n* = 32) and Multiple Sclerosis (3.45%, *n* = 12).
- Cortical Focus: The PFC is the predominant region of interest, mentioned in 18.39% (*n* = 64) of the keyword-based records, emphasizing the focus on executive control in both healthy and pathological gait.
- The high overlap between the ‘Dual-Task’ paradigm and ‘Older Adults’ or ‘Parkinson’s Disease’ clusters indicates that fNIRS is predominantly utilized to evaluate the neural correlates of mobility limitations and the efficacy of neurorehabilitation in these vulnerable populations.

### 3.10. Methodological Landscape

The methodological trends in fNIRS-based gait research exhibit a clear focus on specific cortical regions beyond the frontal lobe and a growing emphasis on technological integration. These methodological categories were derived from keyword frequency analyses and thematic classification of the retrieved records. Based on the keyword analysis, the following methodological pillars were identified:

- Cortical Regions of Interest: While the PFC is the dominant focus, the data reveals a significant interest in motor-related areas. The supplementary motor area (SMA) was identified in 3.45% (*n* = 12) of the records, followed by the motor cortex (*n* = 6) and sensorimotor cortex (*n* = 5). This indicates a shift toward a more comprehensive mapping of the cortical control of walking.
- Multimodal and Immersive Integrations: The integration of fNIRS with other monitoring and stimulus technologies is a prominent trend. VR applications were found in 3.16% (*n* = 11) of the studies, reflecting a push toward ecological validity. Furthermore, the combination of fNIRS with electroencephalography (EEG) (2.87%, *n* = 10) and wearable sensors (1.72%, *n* = 6) highlights the movement toward multimodal data fusion for capturing the complexity of human locomotion.
- Emerging Paradigms: The methodological landscape also includes a focus on motor imagery (2.30%, *n* = 8) and BCI applications (2.01%, *n* = 7), suggesting that fNIRS is increasingly used not only for observation but also as a functional tool in neurorehabilitation and assistive technologies.

This methodological distinction demonstrates that the field is maturing from a PFC-centered approach toward an integrated, multi-region, and sensor-fused methodology capable of addressing the challenges of real-world gait analysis.

## 4. Discussion

### 4.1. Evolutionary Trajectory and Global Networks in fNIRS-Gait Research

The scientometric profile of the 348 retrieved core documents reveals a dynamic and consolidating research domain that has transitioned from an initial exploratory phase into an established methodological framework in human movement science. The calculated compound annual growth rate (CAGR) of 12.53% since 2002 underscores a sustained expansion, with a dramatic inflection point occurring around 2014–2015. In light of the broader neuroimaging literature, this chronological acceleration appears closely associated with the commercial maturation and accessibility of wireless, multi-channel, and lightweight fNIRS instrumentation. Furthermore, the documented average document age of 5.17 years directly quantifies the highly contemporary baseline of the fNIRS-gait literature.

The geographical and institutional distribution of this corpus highlights distinct collaborative architectures across international research hubs. While publication output is led by institutions in the United States—supported by prominent collaborative networks including Yeshiva University, Albert Einstein College of Medicine, Montefiore Medical Center, and the University of Florida—global publication structures exhibit regional variations. Bibliometrically, Asian research exhibits a higher volume of Single Country Publications, whereas European nations demonstrate elevated Multiple Country Publication ratios. While the dataset directly confirms these publication ratios, contextual interpretation from broader policy literature suggests that higher international co-authorship in Europe may reflect established cross-border research frameworks, whereas high domestic volume in other regions indicates strong localized research infrastructure.

From a methodological standpoint, as widely discussed in the movement science literature, fNIRS provides a key advantage over traditional fMRI, which imposes physical immobilization and supine positioning that eliminate postural demands and introduce motion artifacts during locomotion [28,29]. Over the past decade, researchers have aggressively capitalized on fNIRS’s high movement threshold tolerance to establish “ecologically valid” experimental designs that capture brain activity during overground locomotion, obstacle negotiation, and real-world ambulation. The bibliometric data further demonstrates that recent publication growth is no longer sustained solely by isolated fNIRS observations; instead, the frontline of the field has migrated toward multimodal synchronization frameworks, integrating fNIRS with Inertial Measurement Units (IMUs), robotic exoskeletons, and immersive VR environments [30,31,32]. While the bibliometric metrics explicitly quantify the rising prevalence of these technical keywords, broader domain literature interprets this transition as an expansion beyond descriptive neuroscience toward interactive, closed-loop brain–computer interfaces BCI and target-driven neurorehabilitation.

### 4.2. Clinical Landscapes: Stroke, Parkinson’s Disease, and Cortical Compensation Mechanisms

Our quantitative bibliometric dataset directly demonstrates a heavy thematic concentration on healthy aging (27.30%), Parkinson’s disease (10.92%), and stroke (9.20%) across the retrieved core documents. Synthesizing these publication trends with broader clinical literature suggests a widespread scientific interest in conceptualizing human locomotion as a cognitively demanding executive task rather than a purely automated spinal reflex. In a healthy central nervous system, steady-state walking is largely governed by subcortical structures and basal ganglia loops. However, clinical studies referenced within our dataset indicate that when these pathways are compromised, neural control undergoes reorganization. In older adults, the progressive decline in baseline “gait automaticity” necessitates an increased reliance on top-down executive networks to maintain dynamic balance. This compensatory shift is particularly highlighted in FoG paradigms, where neuroimaging studies report PFC hyper-activation during locomotion, reflecting a high cognitive load [33,34,35,36].

Scientometrically, this systemic over-activation of the PFC across vulnerable populations is best contextualized through the Compensation-Related Utilization of Neural Circuits Hypothesis (CRUNCH) framework. According to the CRUNCH model, the aging or impaired brain aggressively recruits auxiliary prefrontal resources during low-demanding or baseline tasks to compensate for structural inefficiencies in primary motor networks [15]. Within the cited clinical literature, this prefrontal engagement is interpreted as a compensatory mechanism that scales inversely with functional independence [37]. Furthermore, intervention studies within our corpus suggest that successful neurorehabilitation—whether mediated through robotic assistance, specialized treadmill protocols, or non-invasive neuromodulation—is frequently accompanied by a structured down-regulation of baseline PFC hyper-activation [4,6], which clinical researchers propose as a potential neural biomarker for improved neural efficiency [38,39,40].

Our bibliometric mapping further identifies “dual-task” paradigms as a central methodological pillar, representing 20.11% of the analyzed fNIRS corpus. While our dataset quantifies the dominant adoption of this paradigm, experimental studies in the literature interpret dual-task walking as a stress-test for prefrontal resource allocation. When executive cognitive tasks are combined with locomotion, clinical populations operating near their neural capacity ceiling often experience severe cognitive-motor interference [41]. As documented across the cited behavioral literature, this executive bottleneck frequently manifests as reductions in stride velocity and elevated gait variability. Ultimately, our scientometric findings map the extensive deployment of fNIRS in these protocols, reflecting its established utility for capturing real-time cortical responses in real-world environments.

### 4.3. Methodological Frontiers: Dual-Task Interferences, Exoskeletons, and Immersive Technologies

The strategic thematic map generated from our corpus highlights an evolving methodological shift where fNIRS is increasingly integrated into immersive, multi-modal research ecosystems. Bibliometrically, the mapping documents strong thematic clusters combining fNIRS with VR, Augmented Reality (AR), and interactive platforms such as the Gait Real-time Analysis Interactive Lab (GRAIL). While our dataset explicitly captures the rising co-occurrence of these technological nodes, broader movement science literature interprets this integration as an effort to maximize ecological validity during ambulatory locomotion, allowing researchers to better explore and potentially evaluate real-time cognitive-motor interference under controlled sensory perturbations [42].

Concurrently, the intersection of fNIRS with robotic neurorehabilitation has established a highly sophisticated platform for monitoring neuroplasticity and cortical modulation. The frequent co-occurrence of terms related to robotic assistance (e.g., Lokomat, Andago, and Ekso systems) reflects an expanding body of research focused on monitoring cortical modulation during task-specific gait training [43,44,45]. Synthesizing these scientometric trends with cited clinical trials suggests that researchers utilize fNIRS to track dynamic resource allocation between the PFC and supplementary motor area (SMA) as mechanical guidance varies [46,47,48]. While our bibliometric data directly quantify the growing volume of exoskeleton-related publications, clinical literature proposes that these integrated paradigms may serve as valuable objective biomarkers for tracking neurorehabilitation outcomes.

The bibliometric convergence toward VR/AR-integrated and robotic-exoskeleton fNIRS paradigms, relative to the comparative marginalization of alternative approaches such as whole-head diffuse optical tomography (DOT) montages or purely resting-state cortical mapping, appears attributable to at least three converging factors evident in the retrieved corpus. First, commercial availability and cost: continuous-wave, sparse-channel fNIRS systems compatible with PFC-centric montages are considerably less expensive and easier to don than dense whole-head DOT arrays, lowering the barrier to entry for the rehabilitation and geriatrics laboratories that dominate the clinical subdomains identified in Section 3.9. Second, translational funding incentives: the clustering of highly cited work around neurorehabilitation outcomes (Section 3.6) and the strong presence of engineering co-authors (Section 3.3) suggest that funding streams targeting closed-loop, device-integrated neurorehabilitation have preferentially rewarded paradigms compatible with real-time decoding (VR, exoskeletons, BCI) over static whole-head mapping, which is better suited to basic-science questions than to intervention studies. Third, computational tractability: dense, whole-head DOT reconstructions require substantially greater modeling and image-reconstruction resources than sparse, PFC-focused channel sets, a burden compounded by the signal-processing heterogeneity discussed in Section 4.4. We note that this explanation is inferred from corpus-level co-occurrence and institutional/funding patterns rather than derived from a direct bibliometric measurement of causality, and it warrants confirmation through dedicated qualitative or survey-based methodological-adoption research.

Despite the immense promise of these technological frontiers, operating neuroimaging modalities within highly dynamic, ambulatory environments introduces severe data-processing bottlenecks that demand robust computational mitigation. The overarching framework of MoBI necessitates the simultaneous ingestion of high-dimensional neural streams (fNIRS and EEG) alongside detailed body kinematics derived from wearable IMUs. To resolve the foundational challenge of millisecond-level multi-device synchronization, the contemporary literature relies heavily on the open-source Lab Streaming Layer (LSL) ecosystem alongside dedicated photodiode triggers to guarantee temporal alignment. Furthermore, to combat motion-related signal degradation intrinsic to continuous overground locomotion, advanced artifact-handling algorithms—including band-pass filtering (BPF), principal component analysis (PCA), and wavelet-based motion artifact reduction (MAR)—have become standard components of the fNIRS processing pipeline [49,50,51,52]. Crucially, to isolate authentic cortical hemodynamic responses from systemic physiological noise, such as scalp dermatological blood flow or heart-rate fluctuations, the integration of short-separation channels (0.8 cm source-detector separation regression) has emerged as a mandatory methodological criterion for ensuring data reliability. The resulting hybrid datasets (fNIRS + EEG + Kinematics) are increasingly decoded through machine learning architectures, where Support Vector Machines (SVM), k-nearest neighbors (kNN), and deep learning Long Short-Term Memory (LSTM) models classify cognitive workload and locomotor intent with high classification accuracies ranging between 80% and 97% [53,54,55]. This analytical progression proves that the field is rapidly transitioning away from retrospective descriptive charting and toward predictive, real-time closed-loop BCI applications.

### 4.4. Methodological Limitations and Future Perspectives

Based on our thematic analysis of the 348 retrieved core documents, the literature highlights several recurring methodological challenges that currently limit the clinical translation of fNIRS in gait research. A primary bottleneck documented across the analyzed studies involves the marked heterogeneity in signal processing pipelines. Divergent transformation variables—such as differential pathlength factors (DPF) and molar extinction coefficients—alongside inconsistent protocols for removing extracerebral systemic noise (e.g., scalp dermatological flow, Mayer waves), complicate direct comparisons across published datasets [49,56,57]. To enhance reproducibility, domain literature emphasizes the need for standardized preprocessing standards, incorporating short-separation regression, automated MAR, and optimized filtering thresholds.

Within the retrieved corpus, short-separation channel (SSC) regression is not applied through a single, uniform algorithm; at least four broad analytical families can be distinguished. (i) Simple nearest-channel subtraction, in which the raw or band-pass-filtered SSC signal is subtracted directly from co-located long-separation channels, is computationally the least demanding but assumes a spatially uniform systemic-noise contribution across the scalp, an assumption increasingly challenged in the literature. (ii) General Linear Model (GLM)-based regression, in which one or more SSC time series are entered as nuisance regressors alongside the task design matrix, is the most frequently implemented approach in toolboxes such as Homer3 and NIRS-SPM and allows the systemic component to be statistically partialled out on a channel-by-channel basis. (iii) Adaptive-filtering approaches (e.g., recursive least squares or Kalman-filter-based SSC regression) dynamically re-estimate the coupling between short- and long-separation channels over time and are theoretically better suited to the non-stationary systemic noise encountered during overground walking, but are computationally more demanding and less widely adopted. (iv) Multivariate decomposition approaches (e.g., PCA- or ICA-based correction informed by SSC data) attempt to isolate a shared systemic component across the full montage rather than channel-by-channel. Critically, the retrieved literature offers no consensus on which of these four families yields the most valid hemodynamic signal during locomotion specifically, since validation studies have been performed predominantly under stationary or seated conditions; the optimal SSC source-detector separation itself (commonly cited between 0.8 and 1.5 cm) also varies by cap manufacturer and has rarely been re-validated for gait paradigms. This absence of a locomotion-specific validation and consensus benchmark is precisely the bottleneck flagged in the Abstract; we clarify here that this constitutes a qualitative synthesis of methodological approaches identified across the corpus rather than a quantitative meta-analytic comparison of their relative performance. Table 2 summarizes these four families for direct comparison.

While the present keyword-based bibliometric methodology was not designed to systematically code software/toolbox mentions across all 348 full-text Methods sections, a qualitative reading of the highly cited subset (Section 3.6) indicates that at least three processing ecosystems dominate the field, each embedding materially different default assumptions. Homer3 (v1.80.2, MATLAB-based, open-source, function-library architecture) requires explicit user specification of motion-correction, band-pass filtering, and SSC-regression steps, maximizing flexibility but also maximizing between-study variability whenever default parameters are incompletely reported. NIRS-SPM (MATLAB-based, built on the SPM neuroimaging framework) instead imposes a General-Linear-Model-centric pipeline with pre-set canonical hemodynamic response functions, trading paradigm flexibility for greater internal standardization, a trade-off not well suited to irregular, self-paced gait events. MNE-NIRS/MNE-Python (open-source, Python-based) integrates fNIRS preprocessing within the broader MNE electrophysiology ecosystem, which facilitates the joint fNIRS-EEG pipelines central to the MoBI trend described in Section 4.3, but remains comparatively new to the gait-analysis community relative to the Homer family. Because none of these ecosystems enforces a mandatory, field-wide default for motion-artifact correction, SSC regression, or the DPF, nominally identical toolbox choices can still yield materially divergent hemoglobin outputs. We agree that this heterogeneity warrants quantitative assessment and identify a systematic, full-text coding of preprocessing pipelines and default parameters across the retrieved corpus—beyond the keyword-frequency scope of the present study—as a priority direction for a dedicated follow-up methodological-scoping review. Table 3 summarizes the three ecosystems’ default assumptions and trade-offs for direct comparison.

Our bibliometric mapping further illustrates that a significant portion of the current literature maintains a PFC-centric focus, often omitting simultaneous monitoring of motor, premotor, and posterior parietal regions. Additionally, a qualitative synthesis of the underlying experimental studies reveals that many pioneering protocols rely on modest sample sizes (e.g., *n* < 20), which may limit statistical power when evaluating subtle cortical changes in heterogenous clinical cohorts [58,59,60,61]. Addressing these limitations in future research will likely require broader multi-center trials and standardized spatial co-registration protocols.

Regarding the specific boundaries of this bibliometric study, utilizing the WoSCC as the sole data source ensures high citation integrity and structured network data, but it may inadvertently omit early-stage engineering developments published in specialized conference proceedings or preprint repositories (e.g., bioRxiv, medRxiv). Independent comparative coverage analyses indicate that WoSCC and Scopus corpora overlap only partially in engineering-adjacent biomedical fields [62], implying that a non-trivial share of engineering-oriented fNIRS-gait records indexed exclusively outside WoSCC may be absent from the present corpus. Future scientometric mappings could synthesize records across multiple indexing platforms (such as Scopus, PubMed, and IEEE Xplore) using cross-database deduplication protocols. Looking forward, our bibliometric trend analysis suggests that the trajectory of fNIRS-gait research is moving toward multimodal sensor fusion. Standardizing MoBI platforms via Lab Streaming Layer (LSL) integration and leveraging machine learning models are promising avenues for real-time cognitive workload decoding [53,63]. Furthermore, optimizing experimental paradigms through short-duration, multi-trial task blocks may improve test–retest reliability, facilitating the integration of fNIRS into personalized neurorehabilitation. Regarding translational validation of the EEG-fNIRS combination highlighted above for Parkinsonian gait, reliable combined EEG-fNIRS animal models remain scarce: rodent 6-hydroxydopamine (6-OHDA) and MPTP-lesioned non-human primate models are well established for probing basal ganglia-cortical circuitry via invasive electrophysiology or fiber photometry, but the thin, highly pigmented rodent scalp and short inter-optode distances pose substantial optical and motion-artifact challenges for translating fNIRS itself to quadrupedal locomotion. Consequently, combined EEG-fNIRS validation work to date has remained almost entirely confined to human bipedal gait, and cross-species translational bridging is an open methodological gap rather than an established pipeline.

## 5. Conclusions

This bibliometric study provides a comprehensive, data-driven synthesis of the global evolution, intellectual structures, and methodological frontiers of fNIRS applications in human gait and locomotion sciences from 2002 to April 2026. By systematically evaluating 348 core peer-reviewed documents, the analysis demonstrates that the field has successfully expanded beyond a tight experimental niche, sustaining a robust annual growth rate of 12.53%. The historical trajectory documents a profound paradigm shift where the scientific community has progressively migrated from highly constrained, stationary surrogate paradigms—such as supine ankle movements or isolated motor imagery tasks—toward ecologically valid, overground walking protocols. Driven by the critical necessity to monitor the ambulatory brain without motion-related signal degradation, fNIRS has established itself as an indispensable tool for mapping the complex transitions between subcortical automatic pathways and top-down cortical executive control, particularly within vulnerable clinical cohorts such as aging populations, stroke survivors, and individuals exhibiting the Parkinson’s disease freezing of gait phenotype.

Looking forward, the maturation of this research domain relies heavily on overcoming the current lack of universal processing pipelines and standardizing multi-modal sensory fusion configurations. To fully exploit the clinical and translational potential of mobile neuroimaging, the international research community must actively transition toward synchronized MoBI frameworks that seamlessly unify fNIRS hemodynamics, electroencephalography, and kinematic sensor data. Overcoming systemic physiological artifacts through short-separation channel regressions and deploying validated machine learning architectures on shared repositories is expected to support the development of baselines required for automated, real-time cognitive workload decoding. Ultimately, bridging the gap between engineering innovations and clinical bedside rehabilitation via standardized, multi-center longitudinal protocols will elevate fNIRS from an evaluative laboratory asset into a definitive, objective biomarker for tracking neuroplasticity, preventing catastrophic falls, and driving personalized, closed-loop neurorehabilitation in real-world environments.

## Figures and Tables

**Table 1:** Top 14 most relevant journals publishing research on fNIRS-based gait analysis.

Sources	Articles	Most Local Cited Sources	H Index	G Index	M Index
Frontiers in Aging Neuroscience	18	265	8	18	0.615
Frontiers in Human Neuroscience	15	510	11	15	0.846
Neurorehabilitation and Neural Repair	15	339	9	15	0.692
Journal of Neuroengineering and Rehabilitation	14	290	10	14	0.769
Neuroimage	13	1839	12	13	0.632
Frontiers in Neurology	12	145	8	12	0.889
IEEE Transactions on Neural Systems and Rehabilitation Engineering	10	138	6	10	0.667
Plos One	10	285	5	10	0.417
Sensors	10	70	8	10	0.889
Brain Sciences	8	98	5	8	0.714
Experimental Brain Research	8	269	3	8	0.167
Gait & Posture	8	518	7	8	0.700
Journals of Gerontology Series A-Biological Sciences and Medical Sciences	7	446	6	7	0.375
European Journal of Neuroscience	6	81	4	6	0.400

**Table 2:** Classification of short-separation channel regression approaches identified across the retrieved fNIRS-gait corpus. This is a qualitative synthesis of methodological approaches, not a quantitative meta-analytic comparison of their relative accuracy.

Family	Underlying Principle	Computational Demand	Adoption Pattern in Corpus	Key Caveat
Simple nearest-channel subtraction	Raw or band-pass-filtered SSC signal is subtracted directly from co-located long-separation channels.	Low	Most accessible; used in earlier and simpler pipelines.	Assumes a spatially uniform systemic-noise contribution across the scalp.
GLM-based regression	One or more SSC time series are entered as nuisance regressors alongside the task design matrix.	Moderate	Most frequently implemented; default approach in Homer3 and NIRS-SPM.	Partials out the systemic component channel-by-channel; validated mainly under stationary or seated conditions.
Adaptive filtering (e.g., recursive least squares, Kalman filter)	Dynamically re-estimates the coupling between short- and long-separation channels over time.	High	Less widely adopted to date.	Theoretically better suited to non-stationary systemic noise during overground walking, but computationally demanding.
Multivariate decomposition (e.g., PCA-/ICA-based)	Isolates a shared systemic component across the full montage rather than channel-by-channel.	High	Emerging; used selectively.	Requires sufficient channel density; no consensus on the optimal number of components.

**Table 3:** Comparison of the three most prominent fNIRS signal-processing toolboxes identified through a qualitative reading of the highly cited subset of the retrieved corpus (Section 3.6).

Toolbox	Platform	Default Preprocessing Approach	Strength	Limitation for Gait Research
Homer3	MATLAB; open-source, function-library architecture.	Requires explicit user specification of motion-correction, band-pass filtering, and SSC-regression steps.	Maximal flexibility.	High between-study variability whenever default parameters are incompletely reported.
NIRS-SPM	MATLAB; built on the SPM neuroimaging framework.	Imposes a General-Linear-Model-centric pipeline with pre-set canonical hemodynamic response functions.	Greater internal standardization.	Canonical hemodynamic-response assumption is poorly suited to irregular, self-paced gait events.
MNE-NIRS/MNE-Python	Python; open-source, integrated within the MNE electrophysiology ecosystem.	Joint fNIRS-EEG preprocessing pipeline.	Facilitates the joint fNIRS-EEG MoBI pipelines described in Section 4.3.	Comparatively new to the gait-analysis community relative to the Homer family.

## Data Availability

The datasets used and/or analyzed during the current study are available from the corresponding author on reasonable request.

## Abbreviations

The following abbreviations are used in this manuscript:

BCI	Brain–Computer Interface
BMI	Brain–Machine Interface
CAGR	Compound Annual Growth Rate
CRUNCH	Compensation-Related Utilization of Neural Circuits Hypothesis
DPF	Differential Pathlength Factor
DT	Dual-Task
EEG	Electroencephalography
fMRI/FMRI	Functional Magnetic Resonance Imaging
fNIRS/FNIRS	Functional Near-Infrared Spectroscopy
FoG	Freezing of Gait
GLM	General Linear Model
ICA	Independent Component Analysis
IMUs	Inertial Measurement Units
MAR	Motion Artifact Reduction
MCP	Multiple Country Publications
MoBI	Mobile Brain/Body Imaging
PCA	Principal Component Analysis
PET	Positron Emission Tomography
PFC	Prefrontal Cortex
QEEG/qEEG	Quantitative Electroencephalography
SCP	Single Country Publications
SSC	Short-Separation Channel
TLA	Three-letter acronym
UK	United Kingdom
USA	United States of America
VR	Virtual Reality
WoSCC	Web of Science Core Collection
